# Prevalence of extended-spectrum beta-lactamase-producing enterobacterial urinary infections and associated risk factors in small children of Garoua, Northern Cameroon

**DOI:** 10.11604/pamj.2020.36.157.21347

**Published:** 2020-07-06

**Authors:** Karyom Djim-Adjim-Ngana, Leila Aïcha Oumar, Brunel Wanda Mbiakop, Hermann Landry Munshili Njifon, Tania Crucitti, Elias Nukenine Nchiwan, Nicolas Njintang Yanou, Louis Deweerdt

**Affiliations:** 1 Department of Biomedical Sciences, University of Ngaoundere, Ngaoundere, Cameroon,; 2 Centre Pasteur of Cameroun Annex of Garoua, Garoua, Cameroon,; 3 Department of Sanitary Engineering and Environment, University of Maroua, Maroua, Cameroon,; 4 Department of Microbiology, University of Yaounde 1, Yaounde, Cameroon,; 5 Centre Pasteur of Cameroun, Yaoundé, Cameroon,; 6 Department of Biological Sciences, University of Ngaoundere, Ngaoundere, Cameroon

**Keywords:** Urinary infection, enterobacteria, antibiotics, synergy test

## Abstract

**Introduction:**

the emergence of extended-spectrum beta-lactamase-producing Entero bacteriaceae (E-ESBLs) is currently a major public health problem in the world and, in particular, in developing countries. In Cameroon, data on E-ESBLs are rare, especially in Garoua and in the northern region of the country. The objective of this study is to document the epidemiology of E-ESBL infections in small children and to explore their associations with possible risk factors.

**Methods:**

this was a cross-sectional, descriptive study conducted from June 14 to September 30, 2018, including small children with suspected urinary tract infections (UTI) attending the outpatient pediatric departments of two health facilities in the city of Garoua. Urine samples were analyzed at the Bacteriology Laboratory of the Pasteur Center of Cameroon, Annex Garoua. Bacterial culture was carried out on Bio-Rad UriSelect® chromogenic agar and the identification was confirmed by bioMérieux API 20E. The antibiotic susceptibility was determined using the bioMérieux ATB UR gallery and the ESBL phenotype was detected by the double disk synergy method according to the CA-SFM 2013 recommendations. The data was analyzed with the R Statistical Software version 2.15.2.

**Results:**

a total of 57 urine samples were collected from children aged from one month to two years, 37 boys and 20 girls. Bacteria were detected by culture in 20 samples: Escherichia coliwas the most frequently (75 %) isolated species followed by Klebsiella pneumoniae(25%). More than half of the infected samples (55%) contained E-ESBL. The presence of an ESBL was significantly associated with previous antibiotic intake up to 3 months prior current UTI (p=0.01664). The E-ESBL strains showed co-resistance to different antibiotics.

**Conclusion:**

this study reveals the important dissemination of E-ESBLs among small children in the community and a high rate of co-resistance to the different antibiotic families commonly used.

## Introduction

Urinary tract infections caused by enteric bacteria producing extended-spectrum beta-lactamases (E-ESBL) are a growing infectious risk and can in many cases lead to therapeutic impasses due to their multidrug resistance [1,2]. This is a very serious public health problem that affects many countries, although resistant strains are often different from one country to another [3]. Currently, the prevalence of E-ESBL varies across countries but appears to be more alarming in low- and middle-income countries (LMIC) than in the rest of the world. Woerther *et al*. estimated that 1.1 billion people were carriers of E-ESBL in South Asia and 110 million in Africa compared with 48 million in the United States and 35 million in Europe [3].

For sub-Saharan Africa, Toudji *et al*. showed in Togo a prevalence of E-ESBLs of 22.4%, consisting of 51.1% *Escherichia coli*and 30.1% *Klebsiella spp*., isolated from pus (47.9%) and urine (40.8%) [4]. In Benin, Zou and Collines, isolated in 342 patients a total of 197 enterobacteriaceae including 143 *E. coli*of which 32/143(22%) were E-ESBL [5]. A study conducted in Niger by Fody et al. reported that 27.7% of multi-resistant isolates of E. coli isolated from different types of biological samples were ESBL-producing strains [6]. In Cameroon, due to the lack of a national multi resistant bacteria surveillance, the extent of circulating E-ESBL strains is unknown. In 2005 Gangoué-Piéboji *et al*. reported in Yaounde 31/259 (12%) of E-ESBL including 12/64 (18.8%) *K. pneumoniae*and 13/91 (14.3%) *E. coli*from urine, pus and blood [7]. Lonchel *et al*. reported a prevalence of 16% faecal ESBL carriage in the community of Ngaoundéré [8]. A recent study on urinary tract infections among women in Yaounde showed that out of 86 isolates of E. coli, 39 strains (45.3%) produced ESBL with prior use of antibiotics as risk factor [9]. The involvement of E-ESBLs in both community and nosocomial urinary tract infections is a real public health problem. Knowledge of the local epidemiological profile of E-ESBLs as well as their current level of resistance to antibiotics is necessary to adapt the treatment guidelines for urinary tract infections to E-ESBL to local epidemiological data. This work represents the first epidemiological study on E-ESBL in the Northern region of Cameroon, with the aim to contribute to the fight against antibiotic resistance and associated risk factors.

## Methods

**Study design**: the study was conducted in the city of Garoua, capital of the North Cameroon region, located between the Adamaoua and Far-North regions of the country. It shares borders with Nigeria in the West and East with the Central African Republic. This was a descriptive and cross-sectional study conducted over a period of four months (from June 14 to September 30, 2018). Small children with suspected urinary tract infections attending two hospitals in the city of Garoua (Garoua Regional Hospital and Hospital of the National Social Welfare Fund of Garoua) were examined by pediatricians. Urine samples were collected and analyzed for diagnostic purposes at the Laboratory of Medical Bacteriology of the Pasteur Center of Cameroon Garoua Annex.

**Laboratory analyzes**: urine was collected using sterile urine collection bags (Urinocol^®^, B. Braun Medical, France) according to the recommendations of the manufacturer (disinfection site sampling, maximum exposure time of 30 minutes). Upon receipt of the sample, the prescribed transport condition was confirmed. Each urine specimen was subjected to a routine urinary cytobacteriological examination comprising: 1) a count of leukocytes and red blood cells in Kova^®^cells which also made it possible to record other elements such as epithelial cells, cylinders, crystals, etc.; 2) a bacterial culture with count of germs (bacteriuria). In accordance with the criteria defined by Kass [10], a urinary tract infection was characterized by leukocyturia (leukocytes>10^4^/mL); and bacteriuria (>10^5^colony forming units (CFU) / mL). Since *E. coli*and *Staphylococcus saprophyticus*are considered specific uropathogens, their pathogenesis threshold is lowered to 10^3^ CFU / mL. The identification of isolated bacteria is based on the use of BioRad^™^type Uriselect 4^®^chromogenic medium (BioRad, France). Enterobacteriaceae are further identified by their morphological and biochemical characteristics (Gram negative bacilli, oxidase negative) completed using BioMérieux^™^Api 20 E^®^galleries (BioMérieux, France). The antibiotic susceptibility was determined by the BioMérieux^™^ATB UR EU^®^gallery (BioMérieux, France) in a semi-solid medium. The ESBL detection was demonstrated by the double disc synergy technique, which consisted of determining synergy between amoxicillin/clavulanic acid (AMC) (BioRad, France) and cefotaxime (CTX) (BioRad, France), ceftazidime (CAZ) (BioRad, France), cefepime (FEP) (BioRad, France) and aztreonam (ATM) ((BioRad, France), the disks of the four last-cited molecules were placed at 30 mm, center to center of the AMC disk on a Mueller Hinton agar plate (BioRad, France) [11]. A positive result was characterized by an ESBL “champagne cork” according to CA-SFM recommendations [12].

**Epidemiological data and statistical analysis**: sociodemographic and epidemiological data were collected during the consultation, using a standardized form. The information collected was: socio-demographic (age, sex) and clinical (clinical signs, anti-arthritis, hospitalizations, etc.) We established a database using Sphinx Plus^2^- Lexica-V5 software including all epidemiological and laboratory data. All variables were examined by univariable analysis using the χ^2^ or Fisher's exact test, as appropriate. All statistical tests were two-tailed. A value of p < 0.05 was considered to be statistically significant. The data was analyzed with the R Statistical Software version 2.15.2 (R Core Team 2012, R Foundation for Statistical Computing, Vienna, Austria) and Excel 2016.

**Ethical clearance**: the study was approved by the Ethics Committee of the University of Ngaoundere decision N° 2018/06/13/UN/R/RFS/CD-DSBM. The study protocol was reviewed and accepted by the authorities of the Hospitals of Cameroon and the Pasteur Center of Cameroon Annex de Garoua. The parents and/or legal guardians of the children provided informed consent. The confidentiality of the information obtained on the subjects of the study was respected.

## Results

**Characteristics of the populationStudy population and demographic characteristics**: during our study, we carried out a total of 57 cytobacteriological urine examinations for diagnostic purposes in small children under the age of 24 months (1 to 19 months). The mean age of the 57 patients was 9.61± 5.1 months; of these, 37 (64.9%) were boys. Among the signs of suspected UTI, 80.7% of the infants showed signs of hyperthermia, 19.3% abdominal pain and 3.5% dysuria. Of the 57 patients, 16 had been hospitalized in the previous year and 29 had received antibiotics within the 3 months prior to inclusion in the study: 13 had received ceftriaxon, 8amoxicillin-clavulinic acid and 7 amoxicillin (Table 1).

**Table 1:** characteristics of the 57 patients included in the study

Characteristics	Study population N=57	Urinary tract infection N=20	P value
**Gender**			
Boys	37	16	
Girls	20	4	0.09163
**Age Median (Interquartile range) (in months)**	9(6-13)	10(2-12)	
**Age range (in months)**			
< 4	10	2	
4-7	7	2	
8-11	20	9	
12-15	10	4	
16-19	9	3	
>20	1	0	0.8377
**Hospital**			
HRG	32	11	
CMS-CNPS/GRA	25	9	1
**Clinical signs**			
Hyperthermia	46	18	
Abdominale pain	7	2	
Dysuria	4	0	0.5853
**Hospitalisation**			
Yes < 48h	6	2	
No	51	18	1
**Current antibiotic treatmetn**			
Yes	13	3	
No	44	17	0.3463
**Antibiotic**			
Ceftriaxone	7	2	
Amoxicillin-Clavulinic acid	5	1	
Amoxicillin	1	0	
Gentamicine	2	1	1
**Previous hospitalisation**			
Yes	16	10	
No	41	10	**0.01229**
**Previous antibiotic use, up to 3 months**			
Yes	29	13	
No	28	7	0.1665
**antibiotic use, up to 3 months**			
Ceftriaxon	13	6	
Amoxicillin-Clavulinic acid	8	4	
Amoxicilline	7	2	
Gentamicin	1	1	0.6964
**Who prescribed the antibiotic?**			
Self medication	6	3	
Professional prescription	23	10	
Not known	28	7	1

**Legend: HRG**: Garoua Regional Hospital; **CMS-SNPS GRA**: Medico-Social Center of the Social Insurance Fund of Garoua

**Proportion of ESBL producers**: bacteria were detected in 20 (35.1%) out of the 57 analyzed urines. In three quarters (N=15) of them *E. coli*was identified, *K. pneumoniae*was identified in the remaining (N=5). ESBL production was observed in 11 (55%) out of the 20 strains of isolated enterobacteriacea. Consequently, we obtained an E-ESBL positivity rate of 19.3% (11/57) among the total study population. The E-ESBLs comprised seven *E. coli*(64%) and four *K. pneumoniae*(36%). Among the isolates of *E. coli*, 7/15 (46.7%) are ESBL producers, compared to 4/5 (80%) among *K. pneumoniae*isolates.

**Characteristics associated with E-ESBL urinary tract infection**: the patient´s characteristics are represented in table 2 according to the presence of ESBL and non ESBL Enterobacteriaceae. Previous use of antibiotics up to 3 months of current UTI was associated with the presence of E-ESBL (p=0.01664) (Table 2).

**Table 2:** univariate analysis of associated risk factors for ESBL

Characteristics	E-ESBL-positive N=11	E- ESBL-negative N=9	P value
**Gender**			
Boys	10	6	
Girls	1	3	0.2848
**Age Median (Interquartile range) (in months)**	9(2-12)	11(2-12)	
**Age range (in months)**			
< 4	1	1	
4 - 7	1	1	
8 -11	6	3	
12 -15	1	3	
16 -19	2	1	
>20	0	0	0.8554
**Hospital**			
HRG	5	6	
CMS-CNPS/GRA	6	3	0.4059
**Clinical signs**			
Hyperthermia	11	7	
Abdominale pain	0	2	0.1895
**Hospitalisation**			
Yes < 48h	1	1	
No	10	8	1
**Current antibiotic treatmetn**			
Yes	1	2	
No	10	7	0.5658
**Antibiotic**			
Ceftriaxone	1	1	
Amoxicillin-Clavulinic acid	0	1	
Amoxicillin	0	0	
Gentamicine	0	1	0.6224
**Previous hospitalisation**			
Yes	8	2	
No	3	7	0.06978
**Previous antibiotic use, up to 3 months**			
Yes	10	3	
No	1	6	**0.01664**
**antibiotic use, up to 3 months**			
Ceftriaxon	5	1	
Amoxicillin-Clavulinic acid	2	2	
Amoxicilline	2	0	
Gentamicin	1	0	0.6224
**Who prescribed the antibiotic?**			
Self medication	3	0	
Professional prescription	7	3	0.528
Not known	1	6	

**Figure 1:**
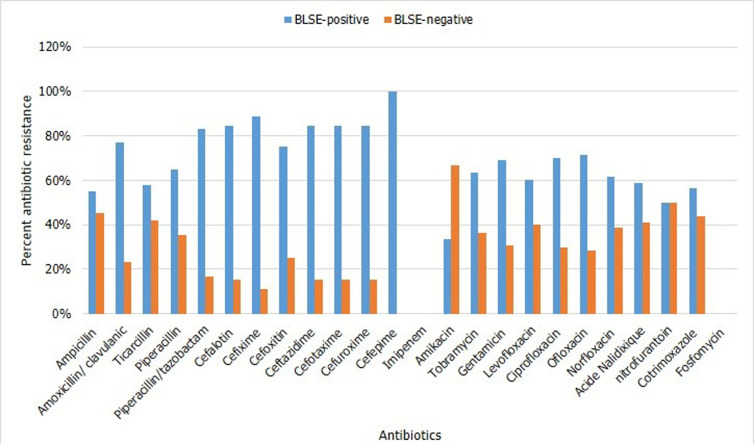
percentage of antibiotic resistance of ESBL-positive and ESBL-negative strains

**Frequency of antimicrobial drug resistance**: Figure 1 shows the antibiotic susceptibility patterns of the isolated E-ESBLs. Most of them were found to have resistance associated with other beta-lactams (up to 100%) but not to imipenem which retained its activity on all isolates. The E-ESBLs were predominantly resistant to gentamycin (81.8%) and tobramycin (63.6%). Among the aminoglycosides, amikacin remains active on all *K. pneumoniae*strains and on 71.4% of *E. coli*strains making it the aminoside of choice when there is a need for antibiotic combination therapy. Similarly, E-ESBLs are predominantly resistant to ciprofloxacin (63.60%) and co-trimoxazole (81.8%). On the other hand, they are mainly sensitive to nitrofurantoin (81.80%) and all are sensitive to fosfomycin.

## Discussion

Our study found an overall high prevalence of uropathogenic E-ESBL (19.3%) among small children with suspected urinary tract infection. This prevalence is little different from that found in other studies, particularly in Morocco by Romli *et al*. (17.5%) and Mohamed *et al*. (12.2%) [1,13], but remains lower to what has been reported by Toudji *et al*. (22.44%) in Togo [4]. Many of these infants have a recent history (less than 3 months) of taking antibiotics. This suggests the role of “antibiotic pressure” in the selection of mutated (multiresistant) strains [14,15]. The mainly plasmid-mediated transmission of genes coding for ESBL is responsible for their rapid dissemination and thus for the increase in the prevalence of ESBL-producing bacteria worldwide [15]. Based on our results, the major ESBL producing strains are *E. coli*and *K. pneumoniae*. This corresponds to the results of several studies in Cameroon [8,16], in other countries in Africa [1,2,4] and in Europe [17] where it has been shown that these two species are most frequently responsible for ESBL production. The frequency of ESBL expression within each enterobacterial species shows a greater ESBL production capacity in *K. pneumoniae* (80.00%) compared to *E. coli* (46.60%). In our series, recent antibiotic therapy, but not prior hospitalization, appeared to be statistically significantly associated with the carriage of an E-ESBL (p =0.01664). The study conducted in Ngaoundere, in a region close to North Cameroon, revealed only the recent antibiotic therapy as risk factor [8], while the study of Mirabeau conducted in Switzerland, reported two risk factors (previous hospitalization and antibiotic use, up to 3 months), in a nososcomial context [17].

Concerning phenotypes resistance to beta-lactams, the isolated E-ESBLs showed resistance to most beta-lactams except imipenem, which remained active for all the strains isolated in this study. However, the absence of E-ESBL carbapenemase producers in our study differs from the results obtained by other researchers (Toudji *et al*. 4.38%; Mohammed *et al*. 3.4%) [1,4]. The resistance of E-ESBLs to many antibiotic classes reduces therapeutic options and maintains a steady increase in the prescription of carbapenems [1]. This phenomenon could be a factor in the appearance of enterobacteria producing carbapenemase. The absence of carbapenemase in our study can be explained by the lack of availability of carbapenems in the North region. We found a high frequency of E-ESBL´s resistant to quinolones. Ciprofloxacin is inactive in 63.6% of the cases, which corroborates with the results of Mohammed *et al*. (92.5%) and Lonchel *et al*. (79.40%) [1,8]. This resistance can be explained by the systematic blind prescription of fluoroquinolones, their availability on the street or the no-compliance of treatment schedules by the patients. Phenicolates and fluoroquinolones flood the markets, available as capsules and tablets, and many are prescribed by the caregiver for all kinds of infections. They are massively used to treat first-line urinary tract infections without prior bacteriological documentation [18]. The use of fluoroquinolones in children is not recommended until the end of their growth period, because of joint toxicity (arthropathies that affect large joints). Exceptions are resistance to all other families of antibiotics or in life-threatening and difficult to treat infections. The frequency of co-resistance of E-ESBL to gentamycin (81.80%) and tobramycin (63.60%) was greater than that of co-resistance to amikacin (18.18%). This is consistent with the results of Mohammed *et al*. and Lonchel *et al*. [1,8]. Amikacin remains the most effective aminoglycoside against E-ESBL [1,8], making it the molecule of choice when an antibiotic of this class is needed [1]. Concerning phenotypes resistance to other antibiotics, fosfomycin retains an intact *in vitro*activity against E-ESBL at 100% which is similar to the studies reported by Mohammed *et al*.[1]. However, fosfomycin cannot be used in very young children.

## Conclusion

This work represents the first community-based study on E-ESBLs in North Cameroon. Our results confirm the high prevalence of these pathogensamong small children with urinary tract infections. Among the E-ESBLs detected, *E. coli*and *K. pneumoniae*were the predominant species. Recent antibiotic therapy was associated with E-ESBL infection, but other environmental factors may be involved. The important co-resistance of E-ESBLs to almost all antibiotic families limits the therapeutic arsenal and increases the risk of treatment deadlock. Our data is important for monitoring the evolution of multidrug resistant bacteria in Cameroon, to improve treatment guidelines and management, and to highlight the need for the implementation of screening methods for ESBL-producing bacteria in routine medical laboratories. Future studies should be extended to other patient populations, and ESBLs should be characterized at molecular level to understand the mechanism of their dissemination. Finally, a national surveillance network for ESBL detection should be put in place as to be able to act timely and prevent further ESBL transmission.

### What is known about this topic

- Urinary tract infection is the second leading cause of heavy use of antibiotics after respiratory tract infections in infants;
- E-ESBLs are present in community settings, the main isolated E-ESBL strains are E. coliand K. pneumoniae.

### What this study adds

- This study found a high overall prevalence of ESBL´s (19.3%), particularly in small children with urinary tract infection;
- The interest of ECBU with antibiogram in the diagnosis and management of bacterial urinary infections;
- Due to the emergence and spread of multiresistant strains, E-ESBL strains show good sensitivity to carbapenems (100%) and fosfomycin (100%).
